# Calibration of the γ-H2AX DNA Double Strand Break Focus Assay for Internal Radiation Exposure of Blood Lymphocytes

**DOI:** 10.1371/journal.pone.0123174

**Published:** 2015-04-08

**Authors:** Uta Eberlein, Michel Peper, Maria Fernández, Michael Lassmann, Harry Scherthan

**Affiliations:** 1 Department of Nuclear Medicine, University of Würzburg, Würzburg, Germany; 2 Bundeswehr Institute of Radiobiology affiliated to the University of Ulm, Munich, Germany; Institut Pasteur, FRANCE

## Abstract

DNA double strand break (DSB) formation induced by ionizing radiation exposure is indicated by the DSB biomarkers γ-H2AX and 53BP1. Knowledge about DSB foci formation *in-vitro* after internal irradiation of whole blood samples with radionuclides in solution will help us to gain detailed insights about dose-response relationships in patients after molecular radiotherapy (MRT). Therefore, we studied the induction of radiation-induced co-localizing γ-H2AX and 53BP1 foci as surrogate markers for DSBs *in-vitro*, and correlated the obtained foci per cell values with the *in-vitro* absorbed doses to the blood for the two most frequently used radionuclides in MRT (I-131 and Lu-177). This approach led to an in-vitro calibration curve. Overall, 55 blood samples of three healthy volunteers were analyzed. For each experiment several vials containing a mixture of whole blood and radioactive solutions with different concentrations of isotonic NaCl-diluted radionuclides with known activities were prepared. Leukocytes were recovered by density centrifugation after incubation and constant blending for 1 h at 37°C. After ethanol fixation they were subjected to two-color immunofluorescence staining and the average frequencies of the co-localizing γ-H2AX and 53BP1 foci/nucleus were determined using a fluorescence microscope equipped with a red/green double band pass filter. The exact activity was determined in parallel in each blood sample by calibrated germanium detector measurements. The absorbed dose rates to the blood per nuclear disintegrations occurring in 1 ml of blood were calculated for both isotopes by a Monte Carlo simulation. The measured blood doses in our samples ranged from 6 to 95 mGy. A linear relationship was found between the number of DSB-marking foci/nucleus and the absorbed dose to the blood for both radionuclides studied. There were only minor nuclide-specific intra- and inter-subject deviations.

## Introduction

DNA double-strand breaks (DSBs) are critical cellular lesions that can result from ionizing radiation exposure [1] but also through other DSB-inducing cytotoxic agents [2,3]. One of the earliest events after DSB formation is the phosphorylation of the histone H2 variant H2AX (encoded by H2AFX [4]) which is then called γ-H2AX since it was first observed in cells irradiated with gamma-rays [1,5,6]. In addition, DSBs also recruit the damage sensor 53BP1 to the chromatin domain surrounding a DSB [7–11] where it co-localizes with γ-H2AX [7,9,12]. In turn, phosphorylation of H2AX is responsible for the accumulation of 53BP1 at the DSB site [13]. Furthermore, 53BP1 facilitates DSB repair by increasing the mobility of the DSB chromatin [14,15]. Therefore, radiation-induced DSBs can be addressed by microscopically visible DNA damage protein foci that display both γ-H2AX and 53BP1. DSB foci disappear by γ-H2AX dephosphorylation after a DSB is repaired [16].

Most *in-vitro* studies of ionizing radiation-induced DSB formation indicate a linear relationship between the number of microscopically visible radiation-induced foci (RIF) and the absorbed dose [1,5]. Blood lymphocytes, for instance, present a dose response of about 9–15 foci/cell per Gy [17–19]. Furthermore, *in-vivo* studies after CT irradiation or radiotherapy show linearity between the dose length product or the total body radiation dose, respectively [19–21]. However, the radiation doses of CT examinations are much smaller compared to the high doses generated in radiotherapy.

Recently, an intercomparison of biodosimetry protocols for γ-H2AX detection [22] revealed that the use of similar protocols by different labs will not result in the same calibration curve after X-ray irradiation. Therefore, it is of great importance that each lab establishes its own calibration curve for its own laboratory standards.

After molecular therapy (MRT) of differentiated thyroid cancer with the isotope I-131 there are only two studies that quantified DSB foci response, using either radiation-induced γ-H2AX and 53BP1 foci [23] or only γ-H2AX foci [24].

Furthermore, there is only a brief description on the induction of radiation-induced DSB foci is a for radiopeptide therapies with Lu-177 [25], a now commonly used molecular MRT [26].

For internal irradiation in MRT, however, the induction and time course of the number of the radiation-induced foci is different compared to external irradiation, since after nuclide incorporation the cells are irradiated not only for seconds or minutes but are continuously irradiated over a longer period with permanently changing dose rate [23].

The aim of this study was to develop a method that allows generating a calibration curve for the DSB focus assay after internal irradiation with radionuclides by creating a low dose and low dose-rate blood irradiation situation *in-vitro*, at dose rates that are similar to the ones that have been observed in nuclear medicine patients.

To this end, we used the two most frequently used radionuclides in MRT, namely I-131 and Lu-177 and quantified the induction of radiation-induced γ-H2AX foci that co-localized with the 53BP1 foci in lymphocytes exposed *in-vitro* and correlated the foci per cell values to the absorbed dose to the blood.

## Material and Methods

### Ethics Statement

The research plan was presented to the ethics committee of the Medical Faculty of the University of Würzburg (Az: 54/13). The ethics committee approved the study by stating that there were no objections to the conduct of the study. All volunteers gave their written consent to participate in the study and for their data to be used for research purposes. The blood was drawn in the Department of Nuclear Medicine of the University Hospital Würzburg, Würzburg, Germany, by experienced physicians of the department. The samples were anonymized for further processing

### Specifications of Radionuclides Used

I-131 and Lu-177 are both beta–emitters with distinct gamma components, which are important for the activity quantification. Their half-lives are 8.023 days and 6.647 days respectively.

I-131 has a γ-emission at 364.5 keV with a high emission probability of 81.2%. The maximum beta energy is 606.3 keV, while the weighted average of the beta decay energy is 181.4 keV.

Lu-177 has two prominent γ-emission lines at 112.9 keV and 208.4 with their respective emission probabilities of 6.2% and 10.4%. The maximum energy of the beta decay is 498 keV and the weighted average of all beta energies is 134.2 keV [27].

### Radioactive Solution Preparation and Activity Quantification

Radionuclide stock solutions (I-131 (Mallinckrodt GmbH, Germany or GE-Healthcare, Germany) and Lu-177 (ITG Isotope Technologies Garching GmbH, Germany)) of known activity were diluted with isotonic NaCl solution (0.9%, B. Braun Melsungen AG, Germany). For exact quantification the amount of activity, a 10 μl aliquot of the radioactive solution was measured in a high purity germanium detector (Canberra GmbH, Germany). The counting efficiency of the germanium detector was determined by repeated measurements of a NIST-traceable standard. Knowing the activity concentrations in the aliquots, the amount of activity needed for achieving absorbed dose rates between 5 and 100 mGy/h to the blood was pipetted into 5 ml round bottom tubes (Sarstedt AG & CO, Germany) and diluted up to 1 ml total volume with NaCl. The lowest and the highest activity were 0.25 MBq and 4 MBq, respectively.

### Blood Sampling and Preparation

In total, nine blood samples ≤ 28 ml were obtained from three healthy test persons (age: 31, 57, 60 years) at different days using Li-Heparin blood collecting tubes (Sarstedt, S-Monovette). Each blood sample was split into 3.5 ml aliquots. One non-exposed sample was used to determine the individual background focus rate. Blood aliquots (3.5 ml) were added to the 1 ml radioactive solution, followed by incubation for 1 h at 37°C on a roller-mixer (35 rpm, Marienfeld GmbH, Germany) to uniformly blend the samples during the exposure time. A 100 μl aliquot of the blood solution was removed to determine the exact activity concentration in the respective blood sample using a germanium detector. After incubation, 3.5 ml of the radionuclide-containing blood solution were filled into a CPT Vacutainer tube (BD, Germany). The white blood cells were separated by 20 minute density centrifugation at 1500 g according to manufacturer’s instructions (BD). Then, the leucocytes were recovered above the interphase and washed twice in phosphate buffered saline (PBS). Ice-cold 99.9% ethanol was added to the cell suspension to result in a solution of 70% ethanol [23].

The fixed white blood cells were stored at least for 24 hours at -20°C and shipped to the Bundeswehr Institute of Radiobiology in Munich, Germany where they were subjected to two-color immunofluorescent staining for γ-H2AX and 53BP1 [12]. Radiation-induced co-localizing γ-H2AX and 53BP1 foci were counted manually in the nuclei of 100 cells using a red/green double band pass filter (Chroma) of a Zeiss Axioobserver 2 epifluorescence microscope by an experienced observer (H.S.). Foci values were expressed as average foci/cell values and the standard deviations were calculated from the experiments assuming a Poisson distribution. The number of radiation-induced damage foci per cell was then obtained by subtracting the background focus rate for each sample.

### Calculation of the Absorbed Dose using a Monte Carlo Simulation

Energy deposition patterns in the blood contained in the vial fully filled with radioactive blood solution were calculated using the radiation transport code MCNPX v2.7 [28] which provides, through the Mesh Tally card type 3, the energy deposited per unit volume and particle.

**Fig 1:**
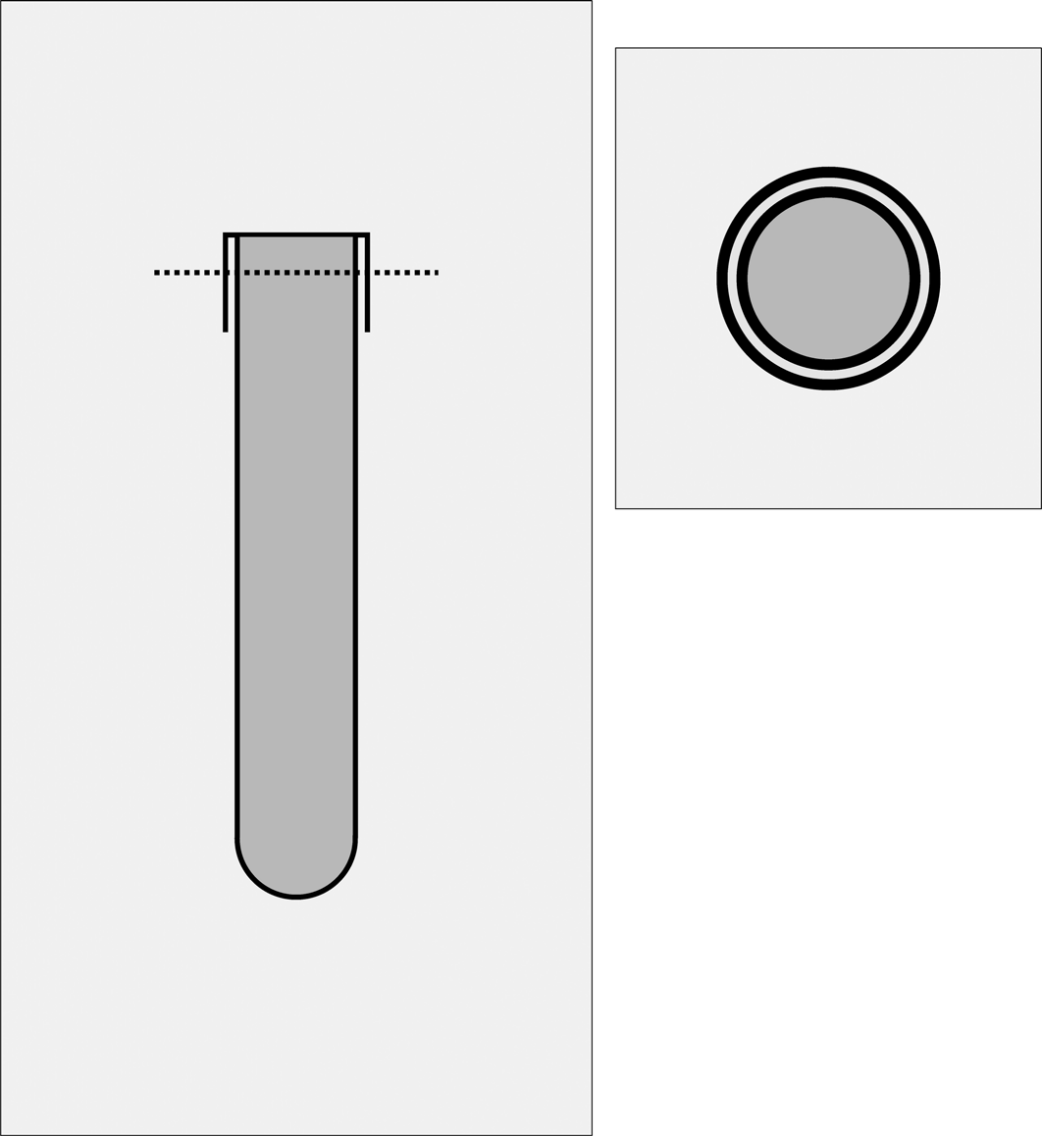
Vial geometry. Schematic drawing showing a longitudinal section (left) and a cross section along the black dashed line drawn on the vial cap (right) of the geometry used for the Monte Carlo simulation. It represents a vial (black) filled with radionuclide-containing blood (dark grey) surrounded by air (light grey).

The set-up for the simulated vial is shown in Fig 1. The left part shows a central plane along the length of the simulated system (vial filled with radioactive blood solution and surrounded by air) and on the right a cross section corresponding to the vial cap. The vial had an internal radius of 0.48 mm and an internal height of 7.49 cm. The radioactive blood solution was considered as a homogeneous mixture of blood with the radionuclide, which was approximated as homogeneous soft tissue-equivalent with density ρ = 1.0 g/cm^3^. The emission of radiation was considered to be isotropic. The vial and its cap were 1.0 mm thick and made of polypropylene (density ρ = 0.9 g/cm^3^). The air surrounding the vial was defined as dry air (density = 1.2 10^-3^ g/cm^3^). 5 cm of air in each direction from the vial external surface was considered. The atomic composition and density of the materials were taken from the STAR (NIST) database [29].

The simulations were performed separately for non-penetrating (betas/electrons) and penetrating (gamma/X-rays) radiation for the used radionuclides I-131 and Lu-177. Their decay data were taken from the publication of Eckerman and Endo [30], which displays the transition energies and probabilities of all emitted particles (including accurately binned beta emission spectra). More details on the simulations are described in a recently published paper by Hänscheid et al. [31], with the only difference that the geometry considered in [31] was a vein portion, while in this publication we consider the geometry of the vial used.

The deposited energies in the vial were converted into the average absorbed dose rate to the blood per nuclear disintegrations occurring in 1 ml of blood, which was denoted in this publication as $\overset{⌢}{\text{S}}$-value (units: Gy·s^-1^·Bq^-1^·ml).

### Statistics

Origin (Version 9.1G, Origin Lab Corporation) was used for data analysis, plotting, and statistical evaluation. For testing whether the data were distributed normally, the Shapiro-Wilk test was used. The sets of samples of different donors were compared using a one-way ANOVA. For these tests we assumed that all data-sets were independent, although, we took several blood samples from the same test persons but on different days.

## Results

### Monte-Carlo Calculations

The average absorbed dose rates to the blood per nuclear disintegrations occurring in 1 ml of blood ($\overset{⌢}{\text{S}}$) for Lu-177 and I-131 for electrons, for photons and for the sum of both contributions are shown in Table 1. Table 1 also displays the average absorbed dose (D) per nuclear disintegrations occurring in 1 ml of blood after 1 h incubation at 37°C.

The data obtained show that, the contributions of photons to the total absorbed dose is less than 3.8% for I-131 and less than 0.6% for Lu-177 (Table 1).

**Table 1:** Average absorbed dose rates to the blood per nuclear disintegrations occurring in 1 ml of blood ($\overset{⌢}{\text{S}}$) and the average absorbed dose to the blood per nuclear disintegrations occurring in 1 ml after incubating the blood for 1 h (D).

	$\overset{⌢}{\text{S}}$(Gy·s^-1^·Bq^-1^·ml)			D (mGy/MBq), 1 h incubation
	Electrons	photons	total	total
Lu-177	2.31 10^-11^	1.32 10^-13^	2.32 10^-11^	83.34
I-131	2.94 10^-11^	1.11 10^-12^	3.05 10^-11^	109.6

### DNA Damage Foci

Blood samples (n = 55) taken from three volunteers (TP1-TP3) were evaluated for DSB formation by counting co-localizing γ-H2AX/p53BP1 damage foci in lymphocytes of blood samples exposed to the isotopes I-131 and Lu-177 in solution. The actual absorbed doses to the blood ranged from 6 to 95 mGy and the average number of RIF/cell from 0.01 to 1.48 per cell. The background number of foci/cell had a mean value of (0.17±0.04) foci/cell ranging from 0.10–0.25 foci/cell.

**Fig 2:**
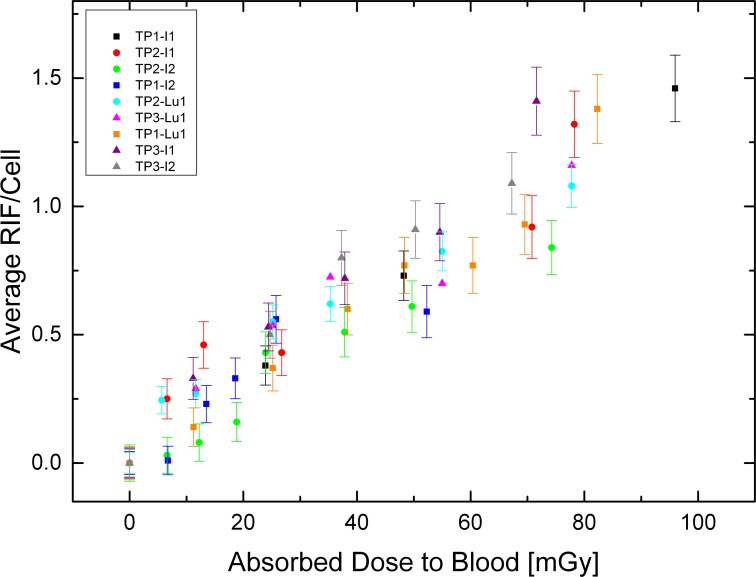
Average RIF/cell as a function of the absorbed dose. Graph showing the results of the individual measurements for our test persons’ blood samples (TP1-TP3) treated with I-131 (I) and Lu-177 (Lu). The error bars represent standard deviation of each single lymphocyte sample, assuming a Poisson distribution.

We determined the RIF per cell as a function of the absorbed dose to the blood for each test person and measurement only minor nuclide-specific, intra- and inter-subject deviations between I-131 and Lu-177 were observed (Fig 2).

Linear fits for each data set were performed separately and for the slopes a normal distribution could be verified using the Shapiro-Wilk test (Origin) with a p-value of 0.30. The mean value of all slopes was (0.0150±0.0018) RIF/cell mGy^-1^.

**Fig 3:**
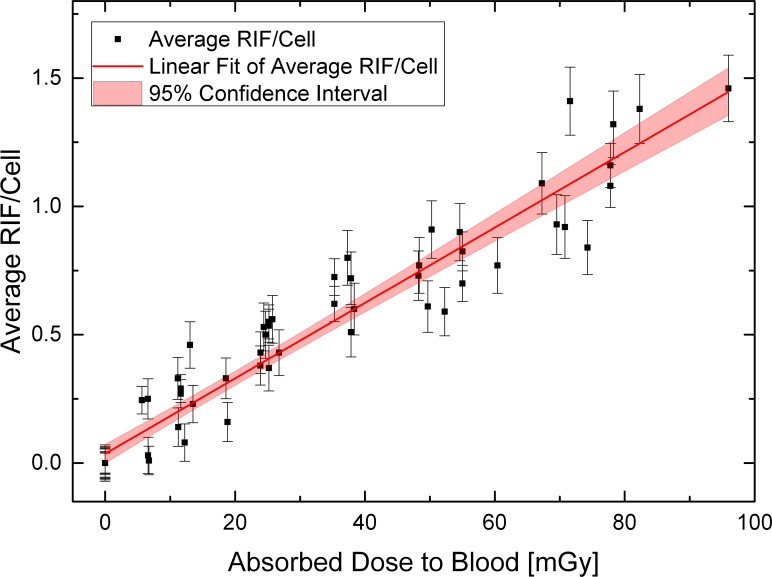
In-vitro calibration curve. Graph showing the calibration curve for our complete pooled data set using blood samples with different dilutions of I-131 and Lu-177. The band indicates the 95% confidence interval.

The set of measurements of the test persons TP1, TP2 and of TP3 was also in agreement with a normal distribution as indicated by the Shapiro-Wilk test and their respective p-values 0.66, 0.29 and 0.29. A one way ANOVA was performed to see if the mean values of the slopes of TP1, TP2 and of TP3 belong to the same basic population. The test showed that there was no significant difference between the mean values of each test person (p = 0.07); therefore, we pooled all data to obtain a single linear fit for all measurements (R^2^ = 0.92):

$$\text{y=0}.0363\cdot \text{RIF/cell+0}.0147\cdot \text{RIF/cell}\cdot {\text{mGy}}^{\text{-1}}\cdot \text{x}$$

y denotes the number of RIF/ cell and x the absorbed dose to the blood in mGy. The standard error of the y-axis intercept is ±0.0182 RIF/cell and the standard error of the slope is ±0.0006 RIF/cell mGy^-1^. The resulting calibration curve for our experiments including a 95% confidence interval is shown in Fig 3.

## Discussion

Here, we undertook a first calibration of the DNA damage focus assay for internal irradiation of whole blood with isotopes in solution at low dose rates. Our calibration measurements simulate dose-ranges of absorbed doses to the blood that have been observed in the first 4–5 h after MRT with I-131 and Lu-177 [32,25].

For this study we choose an incubation time of 1 hour in the presence of dissolved radionuclides. With this setup we aimed to keep the ratio of sample preparation time (1 h) to the incubation time as low as possible for all samples. Longer incubation times would not have allowed us to prepare more than 4 samples for one measurement at the same time, which would have reduced the accuracy of our calibration curve. Additionally, longer exposure times, would have led to a reduction of foci numbers by the progression of DNA repair [1]. Furthermore, we observed that keeping the radionuclide-containing blood samples for too long (>1.5 h) resulted in the stickiness and loss of lymphocytes during the isolation procedure, which made a reliable RIF analysis at advanced time points nearly impossible.

Moreover, shorter incubation times would have required higher activities to deliver the same absorbed doses to the blood, which would have exceeded the activities allowed to handle by our laboratory and additionally would have complicated the handling of several probes simultaneously.

Presently, most published calibration curves for the γ-H2AX focus assay in the lower dose range (below 500 mGy) were obtained by external X-ray irradiation with high dose rates (up to 1.7 Gy/min) [17,33,34,18,35–37,22].

Beels et al. [18] describe the use of external photon-irradiation with an X-ray unit (min ≤ 200 mGy at 20 mGy/min, ≤ 500 mGy at 40 mGy/min) and Co-60 gamma-rays, however, with lower dose rates up to 200 mGy (≤ 200 mGy at 12 mGy/min, ≤ 500 mGy at 300 mGy/min).

At absorbed doses below 10 mGy Beels et al. showed a steep increase of the number of RIF/cell values as a function of the absorbed dose to the blood; while above 10 mGy a shallower slope was observed for the linear fit of the RIF/cell values [18,34,37]. Interestingly enough, this observation was not in agreement with the data of the same authors obtained by external Co-60 gamma-irradiation that showed a linear curve for the complete range of absorbed doses from 1–500 mGy. The calibration curves of Beels et al. in the dose range of 10–500 mGy result in 5.5 RIF/cell for X-rays and 4.3 RIF/cell for the Co-60 irradiation (after irradiation incubation at 37°C for 15 minutes and cooling for 15 minutes to arrest the DNA DSB repair process) [18].

The authors explain this finding by the different track lengths of the secondary particles from the X-ray (100 kVp) exposure compared to the longer track lengths from the high energy gamma-rays from Co-60 (1173 keV and 1332 KeV [27]). Consequently, the X-ray irradiation result in a higher local ionization density.

For our data, we did not obtain two different dose response curves within the dose range investigated (6 to 95 mGy) opposed to the X-ray findings reported by Beels et al. [18]. One explanation could be that, in our experimental set-up, we irradiated the blood mainly with electrons (see Table 1), whereas Beels et al. [18] used either photons of their X-ray unit or gamma irradiation with a Co-60 source, with the latter inducing a linear dose response. In our experimental set-up, I-131 has a maximum beta energy of 606 keV [27] and Lu-177 of 498 keV [27]. Our particle energies are thus just in-between the photon energies applied by Beels et al. [18], which could explain the absence of different RIF induction in the lower dose range.

Rothkamm et al. [17] studied two different tube voltage sets and different dose rates. They chose 150 kV (min ≤ 100 mGy at 83 mGy/min, ≤ 1000 mGy at 160 mGy/min) and 240 kV (min ≤ 100 mGy at 190 mGy/min, ≤ 1000 mGy at 400 mGy/min) and incubated the blood after irradiation for different time periods. There was no difference between the different tube voltages and no deviation of the slope at doses below 10 mGy. At an absorbed dose of 500 mGy the foci numbers after 30 min of exposure (7.0 RIF/cell) are very close to the value that we obtain by extrapolating our calibration curve to 500 mGy (7.4 RIF/cell).

Löbrich et al. [19] irradiated lymphocytes in vitro with 90 kV at a dose rate of 70 mGy/min and observed 4.5 RIF/cell at 30 min after X-ray exposure of 500 mGy.

In a recent study by Vandevoorde et al. [38] the cord blood of three healthy newborns was irradiated up to 500 mGy with a 100 kVp X-ray tube. The samples were kept for 30 minutes at 37°C for DNA damage signaling at then the repair was arrested by cooling the samples to 0°C for 15 minutes. The foci analysis at 500 mGy resulted in 5.7 RIF/cell. In the very low dose range (0–12 mGy) the authors found a foci dose-response curve comparable to that of Beels et al. [18] the data points being lower, however. Hence it may be that the cooling step induces technical variation.

Scherthan et al. [39] used the same method as in this work for fixing and staining of the cells, but with a different observer analyzing the cells. X-ray irradiation with 500 mGy at 0.5 Gy/min with a tube voltage of 220 kVp resulted in 4.2 γ-H2AX RIF/cell 30 minutes after exposure.

As already mentioned there are variations in laboratory methods and standards of the focus assay. In addition, there are operator-specific differences when it comes to counting DSB foci. This definitely affects the results of the evaluation. Moreover, most of the research groups performed direct staining of the cells, while we fix our cells in ethanol and can stain the cells for DSB foci. Furthermore, we apply a double band pass filter for DSB-marking γ-H2AX/53BP1 foci.

As far as a potential age-dependency of the results is concerned no such effect was observed in the aforementioned studies. While in this study blood samples of volunteers of different ages were drawn, the foci results were very similar. The study by Roch-Lefèvre et al. [35] identified five individuals with a higher background foci level, however, any age- or gender-related effects were not observed. Therefore, we would not expect, for our *in-vitro* study in adults, an age or gender dependency of the γ-H2AX foci assay. In agreement, our limited sample of volunteers did not show such effects.

As far as the intra- and inter-subject variability and the isotope dependency of our results are concerned, we are aware of the fact that the statistical tests we performed are limited by the low number of healthy individuals enrolled in this feasibility study. However, most of the studies with external exposure were also performed either with three volunteers only [18,38] or the number of scored individuals per data point was not directly obvious from the study [17,19,35] or even only one volunteer [33] was considered. Furthermore, in our study we show the single data points for each measurement and test persons, whereas, in most studies the data is directly pooled [19,17,34,18,38] and the individual foci trend is, therefore, not apparent.

For a more accurate study more volunteers are needed for each radionuclide and no volunteer should be used twice for donating blood to ensure completely volunteer-independent results.

## Conclusion

In this work we described a novel method for the calibration of the γ-H2AX/53BP1 DNA damage focus assay for radionuclide exposure in solution and provide first results on the relationship between the number of radiation-induced DSB foci and well-defined absorbed doses to the blood using an in solution radionuclide exposure model.

The presented set-up will help us to understand and further improve the dose calculation method for *in-vivo* experiments using blood samples from patients after radionuclide therapies.

Moreover, this calibration curve in conjunction with the DNA damage focus assay could be used as a quick tool to obtain knowledge on the corresponding absorbed dose to the blood when an individual was irradiated in a radiation accident or a malevolent action. With this approach it would be possible to more adequately adjust the medical treatment of people involved in radiation accident.

## Supporting Information

S1 File Table A. Test persons' specific counts for I-131. Table B. Test persons' specific counts for Lu-177.
(PDF)
