# Supplementary material for: Calibration of the γ-H2AX DNA Double Strand Break Focus Assay for Internal Radiation Exposure of Blood Lymphocytes
Source: PLoS One. 2015 Apr 8;10(4):e0123174. doi: 10.1371/journal.pone.0123174 (PMC4390303; doi:10.1371/journal.pone.0123174)
Supplement: S1 File — Table A. Test persons' specific counts for I-131. Table B. Test persons' specific counts for Lu-177. (PDF) [file pone.0123174.s001.pdf]

Calibration of the γ-H2AX DNA double strand break focus assay for internal radiation exposure of blood lymphocytes

Uta Eberlein[1], Michel Peper[2], Maria Fernández[1], Michael Lassmann[1,¶], Harry Scherthan[2,¶]
1 Department of Nuclear Medicine, University of Würzburg, Würzburg, Germany
2 Bundeswehr Institute of Radiobiology affiliated to the University of Ulm, Munich, Germany

¶ML and HS are joint senior authors
∗ E-mail: eberlein_u@ukw.de

Supporting Information

Table A. Test persons' specific counts for I-131

I-131			
Patient ID	Absorbed Dose to Blood [mGy]	Average RIF/Cell	Standard Deviation
TP1-I1	0.00	0.00	0.03
	23.89	0.38	0.076
	48.24	0.73	0.10
	95.96	1.48	0.13
TP1-I2	0.00	0.00	0.04
	6.73	0.01	0.06
	13.49	0.23	0.07
	18.58	0.33	0.08
	25.74	0.56	0.09
	52.28	0.59	0.09
TP2-I1	0.00	0.00	0.06
	6.57	0.25	0.08
	13.04	0.46	0.09
	26.75	0.43	0.09
	70.79	0.92	0.12
	78.25	1.32	0.13
TP2-I2	0.00	0.00	0.05
	6.58	0.03	0.07
	12.24	0.08	0.07
	18.84	0.16	0.08
	23.92	0.43	0.08
	37.84	0.51	0.10
	49.69	0.61	0.10
	74.29	0.84	0.11
TP3-I1	0.00	0.00	0.06
	11.16	0.33	0.08
	24.40	0.53	0.09
	37.85	0.72	0.10
	54.59	0.90	0.11
	71.60	1.41	0.13
TP3-I2	0.00	0.00	0.06
	24.69	0.50	0.09
	37.29	0.80	0.11
	50.28	0.91	0.11
	67.26	1.09	0.12

Table B. Test persons' specific counts for Lu-177

Lu-177			
Patient ID	Absorbed Dose to Blood [mGy]	Average RIF/Cell	Standard Deviation
TP1-Lu1	0.00	0.00	0.06
	11.24	0.14	0.07
	25.19	0.37	0.09
	38.34	0.60	0.10
	48.36	0.77	0.11
	60.40	0.77	0.11
	69.54	0.93	0.12
	82.29	1.38	0.13
TP2-Lu1	0.00	0.00	0.03
	5.65	0.25	0.05
	11.63	0.27	0.05
	25.19	0.55	0.07
	35.29	0.62	0.07
	55.00	0.83	0.08
	77.79	1.08	0.08
TP3-Lu1	0.00	0.00	0.04
	11.63	0.29	0.05
	25.19	0.54	0.06
	35.29	0.73	0.07
	55.00	0.70	0.07
	77.79	1.16	0.09
